# Cosmetic Outcome Assessment following Breast-Conserving Therapy: A Comparison between BCCT.core Software and Panel Evaluation

**DOI:** 10.1155/2014/716860

**Published:** 2014-09-22

**Authors:** Max Hendrik Haloua, Nicole Marianna Alexandra Krekel, Gerrit Johannes Albertus Jacobs, Barbara Zonderhuis, Mark-Bram Bouman, Marlon Eugène Buncamper, Franciscus Bernardus Niessen, Henri Adolf Hubert Winters, Caroline Terwee, Sybren Meijer, Monique Petrousjka van den Tol

**Affiliations:** ^1^Department of Surgical Oncology, VU University Medical Center, De Boelelaan 1117, 1081 HV Amsterdam, The Netherlands; ^2^Department of Plastic and Reconstructive Surgery, VU University Medical Center, De Boelelaan 1117, 1081 HV Amsterdam, The Netherlands; ^3^Department of Epidemiology and Biostatistics and the EMGO Institute for Health and Care Research, VU University Medical Center, De Boelelaan 1117, 1081 HV Amsterdam, The Netherlands

## Abstract

*Purpose*. Over recent decades, no consensus has yet been reached on the optimal approach to cosmetic evaluation following breast-conserving therapy (BCT). The present study compared the strengths and weaknesses of the BCCT.core software with a 10-member panel from various backgrounds. *Methods*. Digital photographs of 109 consecutive patients after BCT were evaluated for 7 items by a panel consisting of 2 breast surgeons, 2 residents, 2 laypersons, and 4 plastic surgeons. All photographs were objectively evaluated using the BCCT.core software (version 20), and an overall cosmetic outcome score was reached using a four-point Likert scale. *Results*. Based on the mean BCCT.core software score, 41% of all patients had fair or poor overall cosmetic results (10% poor), compared with 51% (14% poor) obtained with panel evaluation. Mean overall BCCT.core score and mean overall panel score substantially agreed (weighted kappa: 0.68). By contrast, analysis of the evaluation of scar tissue revealed large discrepancies between the BCCT.core software and the panel. The analysis of subgroups formed from different combinations of the panel members still showed substantial agreement with the BCCT.core software (range 0.64–0.69), independent of personal background. *Conclusions*. Although the analysis of scar tissue by the software shows room for improvement, the BCCT.core represents a valid and efficient alternative to panel evaluation.

## 1. Background

### 1.1. Breast-Conserving Therapy

Breast cancer treatment has changed dramatically over the past few decades. In many countries breast-conserving therapy (BCT), that is, breast-conserving surgery combined with postoperative radiotherapy, has become the standard of care for early-stage breast cancer [1–5]. BCT was primarily developed to decrease morbidity and improve cosmetic outcome without compromising oncological outcomes, factors that may be crucial to patient satisfaction and quality of life [6–8].

However, fair to poor cosmetic outcomes following BCT are still observed in up to 33% of patients undergoing BCT [6–9]. As a consequence, new surgical techniques (oncoplastic and/or ultrasound-guided surgery) and radiotherapeutic strategies (i.e., boost dose) have been developed to improve cosmetic outcomes and lower patient morbidity [10–12]. These new treatment modalities should now be evaluated using reliable, time efficient, and reproducible cosmetic evaluation tools.

Despite the fact that factors influencing cosmetic outcome have been under evaluation since the 1980s and that various subjective and objective evaluation techniques have been studied, consensus has not yet been reached on optimal approaches to cosmetic evaluation and the development of comparable scoring methods [13–15].

#### 1.1.1. Subjective Evaluation Methods: Patient Self-Evaluation and Panel Evaluation

Of the many subjective evaluation methods that are available, patient self-evaluation is valuable due to the central role of the patient's subjective experience in the assessment of quality of life. Drawbacks associated with patient self-evaluation include dependence on several factors that are not amenable to quantification, such as age and socioeconomic status, and the consistent reporting of better scores by patients than by professionals [16].

Panel evaluation remains the most common and accepted approach to the subjective evaluation of cosmetic results, and the approach also takes breast asymmetry, scars, and skin changes into account [13, 15]. Using photographs of the breasts, panel evaluation rates a range of aspects and generally uses the so-called 4-point Likert or Harvard scale, with classification of overall cosmetic outcome as excellent, good, fair, or poor [16–18]. Because strong variation between observers is common, a panel should consist of at least 5 members, including both professionals and nonprofessionals from diverse backgrounds [16]. Despite the widespread use of panels, the interobserver reliability of different panel constitutions (e.g., panels including observers from different backgrounds and specialisations) and the validity of such panel evaluations remain unclear.

#### 1.1.2. Objective Evaluation Methods: BCCT.core

When assessing objective evaluation methods, the key parameter in analysing cosmetic results appears to be the assessment and measurement of asymmetry, in which the ultimate cosmetic objective of BCT is the attainment of two identical breasts [15]. Objective computerised methods have been developed with this goal in mind. These include the recently developed breast cancer conservative treatment.cosmetic results (BCCT.core) software, which provides an extensive set of automated measurements that cover a broad range of items that reflect overall cosmetic outcome [14]. Using digital marks on the nipples, axillae, and sternum jugular notch, this software automatically identifies the breast contour and carries out automated measurements including breast shape, breast volume, deformity, nipple position, scar visibility, and skin changes. Using this range of items and a 4-point scale, the results reflect cosmetic issues that may arise following BCT and allow overall assessment of cosmetic outcome [19].

The claimed advantages of the BCCT.core software compared to panel evaluation include the fast and accurate reporting of results that were previously very time-consuming. In addition, a reliable and automated approach to the assessment of cosmetic outcomes would enable comparison of results from different breast surgery units worldwide [20].

### 1.2. Aims of the Study

In this study, the level of agreement between BCCT.core software and panel evaluation was evaluated by comparing both the overall scores and the specific items in each evaluation method (e.g., volume, scar, and skin). The interobserver reliability of various panel constitutions (in order to assess the influence of background and specialisation) and intraobserver reliability of the individual panel members were also investigated.

## 2. Methods

### 2.1. Patients

A total of 109 consecutive patients who had undergone BCT from January to November 2006 were included in this study. All patients underwent BCT for T1-T2 breast cancer and were photographed after at least a 1-year follow-up period measured from the beginning of the treatment. Patients who underwent previous surgery of the breasts and those who previously had radiation of the chest region were excluded from the study. Patient, tumour, and treatment characteristics (such as radiotherapy, type of axillary surgery, weight, and volume of the specimen) were collected from hospital records and written informed consent was obtained.

Breast surgery consisted of palpation-guided lumpectomy for palpable tumours and wire- or ultrasound-guided lumpectomy for nonpalpable tumours. Axillary surgery consisted of either a sentinel node procedure (SN) or an axillary lymph node dissection (ALND). All patients received radiation therapy of the whole breast and a radiotherapy boost to the tumour bed, where indicated. Adjuvant chemotherapy or hormonal therapy was administered depending on the tumour characteristics of the patient and according to national guidelines.

### 2.2. Photographs

Digital frontal photographs of the breasts, including the suprasternal notch, were taken at a mean follow-up time of 20 months (range 12–40 months). All patients were photographed in a standardised manner, with their arms at their sides, allowing meaningful comparison between patients. All photos were taken by a single photographer using a RICOH Caplio R3 5.0 megapixel digital camera. Photographs were then compiled into a PowerPoint presentation for panel evaluation.

### 2.3. Panel Scoring

All photographs were scored by a 10-member panel consisting of two experienced breast surgeons (male and female, at least 10 years of experience with breast cancer surgery), two surgical residents (both female), two laypersons (male and female), and four experienced plastic surgeons (all male, at least 10 years of experience with breast reconstruction surgery). The evaluation took place in June 2013. Cosmetic scoring was performed using a digital video projector. All members of the panel were blinded to each other. Twenty randomly selected photographs (not included in the actual study) were shown to the panel before scoring began in order to avoid skewness between observations. The panel scored various topics on a four-point Likert scale as described in Table 1. Intra-observer reliability was tested by once again evaluating a set of 50 randomly selected photographs at 4 weeks after the first evaluation.

A questionnaire was used to evaluate the cosmetic outcome of the treated breast compared to the untreated breast for seven items using the 4-point Likert scale, based on the questionnaire described by Aaronson et al.: I: breast shape; II: breast volume; III: breast deformity; IV: nipple position; V: appearance of the surgical scar; VI: skin alterations; and VII: overall cosmetic result [14].

### 2.4. BCCT.core

The BCCT.core software (version 20) not only incorporates a broad set of automatic asymmetry calculations, including scar and skin changes, but also provides an overall cosmetic outcome on the same 4-point Likert scale used in the panel evaluation. The frontal photographs of the 109 patients were analysed in September 2013 according to the BCCT.core manual. The investigator digitally marked the following points: the nipples, axillae, and sternum jugular notch. The BCCT.core software then automatically identified the breast contour for further automated calculations. These dimensionless asymmetry calculations include the following:** pBRA** (the relative breast retraction assessment), quantifying the relative difference in nipple position between both breasts;** pUNR** (the relative upward nipple retraction), quantifying the relative difference between nipple levels;** pBCE** (the relative breast compliance evaluation), quantifying the relative difference between the left and right nipple to inframammary fold distance; and** pBAD** (the relative breast area difference), quantifying the relative difference between areas of the left and right breasts [21].

Colour difference and scar visibility were also evaluated with the BCCT.core software, each with 8 different variables as described by J.S. Cardoso and M.J. Cardoso [22]. Finally, the software generated an overall score on the same 4-point Likert scale as used by the panel (Table 1).

### 2.5. Statistical Analysis

All analyses were performed with IBM Statistical Package for the Social Sciences (version 20.0; IBM, Armonk, NY, USA). Patient, tumour, and treatment characteristics were compared using Student's *t*-test and Chi-square statistical tests.

In order to assess the agreement of the BCCT.core overall score with the mean 10-member panel score, the weighted kappa was used. Calculation of the weighted kappa was performed in SPSS using the intraclass correlation coefficient (ICC, two-way random effects model), which has the same value as the quadratic weighted kappa [22, 23]. Inter- and intraobserver variability among the panel members were also calculated using the weighted kappa method. Landis and Koch characterised values of 0–0.20 as slight agreement, 0.21–0.40 as fair, 0.41–0.60 as moderate, 0.61–0.80 as substantial, and 0.81–1 as almost perfect [24].

Weighted kappa was subsequently calculated for different subgroups of raters, initially excluding laypersons, then excluding laypersons and residents, and finally solely with plastic surgeons, thereby allowing differences in reliability within subgroups of the panel by speciality and experience to be assessed. Percentage of absolute agreement was also calculated. In order to compare the BCCT.core with the panel on a 4-point Likert scale, the mean overall cosmetic score of the 10 panel members was calculated and rounded to the nearest integer.

Spearman's correlation and Pearson correlation were used to assess the correlation of the mean score of different panel items to automated calculations of the BCCT.core software. This was only applicable for certain panel items that matched a calculated score of the software (e.g., panel item nipple position and the dimensionless feature pBRA). The absolute values of the correlations can be interpreted as very weak (0–0.20), weak (0.21–0.40), moderate (0.41–0.60), strong (0.61–0.80), and very strong (0.81–1).

## 3. Results

### 3.1. Patient and Tumour Characteristics (Table 2)

The average age of the 109 patients included in this study was 57.8 years (range 36–83), at the time of the operation. Of the tumours, 72 (66%) were palpable and 37 (34%) were nonpalpable, which resulted in 64 (59%) palpation-guided excisions, 17 (15%) wire-guided excisions, and 28 (26%) ultrasound-guided excisions. In 58 cases (53%) tumours were located in the upper outer quadrant, invasive ductal carcinoma was present in 93 cases (85%), all patients received radiotherapy, and radiotherapy boost was administered in 92 cases (84%). Nineteen patients (17%) had to undergo a reexcision for tumour-involved margins.

### 3.2. Cosmetic Outcome and Inter- and Intraobserver Agreement (Table 3)

#### 3.2.1. Panel Evaluation

The average of the overall cosmetic outcome as evaluated by the whole panel was excellent in 8 patients (7%), good in 46 (42%), fair in 40 (37%), and poor in 15 (14%). Interobserver agreement for the whole panel, calculated using weighted kappa, was 0.66 (95% confidence interval 0.59–0.73). The mean percentage interobserver absolute agreement of the panel, defined as the percentage of scores that were exactly the same between two raters for the overall score, was 48% (range 36–65).

Intraobserver agreement of individual raters, calculated with the use of the weighted kappa, varied between 0.54 and 0.80, suggesting moderate to substantial agreement.

#### 3.2.2. BCCT.core

The overall cosmetic outcome with BCCT.core was excellent in 10 patients (9%), good in 54 (50%), fair in 34 (31%), and poor in 11 (10%).

#### 3.2.3. Panel Evaluation versus BCCT.core

The mean absolute agreement of the BCCT.core software and individual panel members was 47% (range 39–53). The weighted kappa of the overall BCCT.core software score and the average overall panel score per patient was 0.68 (95% confidence interval 0.57–0.77), suggesting substantial agreement.

### 3.3. Subgroup Panel Analysis

#### 3.3.1. Panel without Laypersons

This subgroup panel consisted of 2 surgical residents, 2 breast surgeons, and 4 plastic surgeons. The weighted kappa of the interobserver reliability of the panel was 0.69 (95% confidence interval 0.62–0.75). The weighted kappa of the average score compared with the BCCT.core software was 0.69 (95% confidence interval 0.57–0.77).

#### 3.3.2. Highly Specialised Panel

This panel consisted of 2 breast surgeons and 4 plastic surgeons, and the weighted kappa of the interobserver reliability was 0.67 (95% confidence interval 0.60–0.74). The weighted kappa of the average score of the specialised panel compared with the BCCT.core software was 0.67 (95% confidence interval 0.55–0.76).

#### 3.3.3. Plastic Surgeon Panel

This panel consisted of 4 plastic surgeons; the weighted kappa of the interobserver reliability was 0.70 (95% confidence interval 0.63–0.77). The weighted kappa of the average plastic surgeons' score compared with the BCCT.core software was 0.64 (95% confidence interval 0.52–0.74).

### 3.4. Comparison of Panel Items with Specific BCCT.core Items

#### 3.4.1. Volume

Interobserver agreement of the panel on the item volume was substantial (weighted kappa of 0.61). The average score of the “breast volume” question scored by the panel showed a substantial correlation between with the pBAD (dimensionless Breast Area Difference) of the BCCT.core software, with a Pearson correlation of 0.60.

#### 3.4.2. Nipple Position

Interobserver agreement of the panel on the item nipple position was also substantial (weighted kappa of 0.63). The average score of the nipple position by the panel showed a substantial correlation with the pBRA, pUNR, and pBCE of the BCCT.core software (Spearman's correlation ranging between 0.38 and 0.44).

#### 3.4.3. Skin

Interobserver panel agreement on the appearance of skin was moderate (weighted kappa of 0.47). The average score for skin appearance by the panel showed a substantial correlation with all colour specific items of the BCCT.core software (cX^2^L, cX^2^a, cX^2^b, cX^2^Lab, cEMDL, cEMDa, cEMDb, and cEMDLab) (Spearman's correlation ranging between 0.24 and 0.36).

#### 3.4.4. Scar

Scoring of scar features by the panel also showed moderate interobserver agreement (weighted kappa of 0.45). Low correlations were obtained for the average scar score of the panel with the scar specific items of the BCCT.core (sX^2^L, sX^2^a, sX^2^b, sX^2^Lab, sEMDL, sEMDa, sEMDb, and sEMDLab) (Spearman's correlation ranging between 0.09 and 0.13).

## 4. Discussion

In the absence of a gold standard for cosmetic outcome analysis, panel evaluation has long been considered the most appropriate method of assessing cosmetic outcome [8, 25, 26]. The present study clearly showed that panel evaluation with 10 observers results in a substantial agreement between observers (kappa 0.66). In addition, we showed that the constitution of the panel did not impact on the level of agreement, which indicates that agreement between panel members did not increase with greater experience with breast surgery (e.g., a panel lacking laypersons and surgical residents did not lead to higher levels of agreement).

Due to the substantial workload associated with evaluation by 10 observers, we explored the feasibility of smaller panels using 20 random combinations of 3 observers from the initial database. These panels showed substantial agreement between observers, with a mean weighted kappa of 0.66 (range 0.58–0.73), indicating that a reliable overall cosmetic outcome score can be achieved using a panel with any constitution of 3 observers. This finding contrasts with that of Vrieling et al., who suggested that a panel of at least 5 observers is required [16].

To date, only a limited number of research groups have evaluated BCCT.core software. This software was originally validated against a panel of 12 expert observers (surgeons operating on more than 200 patients per year), in a study with a consensus design, meaning that the software was compared with the observer's score with the highest agreement with the consensus score on 30 cases and not by calculating the mean overall score of 12 expert observers [18, 27]. The BCCT.core software has also been compared with panel evaluation in a study by Heil et al. of overall cosmetic outcome following BCT. These authors reported fair agreement between observers (mean weighted kappa of 0.31) and fair to moderate agreement between the panel and the software (mean weighted kappa of 0.24–0.45) [28].

Because use of BCCT.core software is now being described in an increasing number of studies, it is important to assess the reliability of the software [29–33]. The approach taken in the present study, by investigating specific software items (e.g., skin, scar, and volume) in comparison to the same items when scored by a panel, is the first of its kind and will facilitate understanding of the software.

Our results showed that the overall BCCT.core software score was substantially in agreement with the mean overall panel score for cosmetic outcome (kappa 0.68). Analysis of specific items such as volume, nipple position, and skin showed correlations ranging from very weak to strong between the panel and the software with especially very weak correlations on scar items (between 0.09 and 0.13). Further analyses of the scar specific items showed that patients with marked scarring of the breast received a better overall classification with the BCCT.core than from the panel. This indicates that BCCT.core software does not yet adequately detect marked scarring and that there is room for improvement on scar specific items.

A limitation of the present study was the absence of a comparison between the software and patient self-evaluation. Patient self-evaluation might have provided valuable information not only on items related to breast cosmetic outcomes (subtle retraction or firmness) but also on functional aspects [25]. We suggest that patient self-evaluation should be performed alongside panel or BCCT.core evaluations, as self-evaluation reflects the psychological adaptation of the patient to the appearance of the breast.

With the increasingly widespread use of oncoplastic breast surgery techniques and the accompanying lack of robust studies with good methodology, the evaluation of the reliability and validity of the various techniques for the assessment of cosmetic outcome should now be a priority. Well-designed prospective studies on oncoplastic breast surgery should incorporate the evaluation of cosmetic outcome as standard, with the use of reliable and valid techniques providing the best possible assessment of cosmetic outcome [10]. Although BCCT.core software can provide valid cosmetic outcome scores when compared to a panel evaluation and thus facilitate comparison of different breast surgery units worldwide, the analysis of some items shows a need for improvement when compared to panel evaluation.

The claimed advantages of BCCT.core are the fast and accurate reporting of results and calculations that were previously time-consuming prior to the development of this software [20]. The rapid assessment of results (approximately 3 minutes per photograph) is clearly facilitated by the BCCT.core. Following improvements in the analysis of scar features, the BCCT.core software could represent a valid tool in the assessment of cosmetic outcome.

## Figures and Tables

**Table 1:** Harvard scale (4-point Likert scale).

Excellent	Treated breast nearly identical to untreated breast
Good	Treated breast slightly different from untreated breast
Fair	Treated breast clearly different from untreated breast but not seriously distorted
Poor	Treated breast seriously distorted

**Table 2:** Patient and tumour characteristics.

Patient population	Mean (range)
Patient age at operation (years)	57.8 (36–83)
Tumour size (cm)	1.8 (0.3–5.5)
(i) Palpable	72 (66%)
(ii) Nonpalpable	37 (34%)
Axillary surgery	
(i) SN	73 (67%)
(ii) ALN	36 (33%)
Side	
(i) Left	57 (52%)
(ii) Right	52 (48%)
Type of carcinoma	
(i) Ductal	93 (85)
(ii) Lobular	5 (5%)
(iii) Other	11 (10%)
Quadrant	
(i) Lateral Upper Quadrant	58 (53%)
(ii) Lateral Lower Quadrant	17 (15%)
(iii) Medial Lower Quadrant	5 (5%)
(iv) Medial Upper Quadrant	24 (22%)
(v) Periaureolar	4 (4%)
(vi) Missing	1 (1%)
Boost	
(i) Yes	92 (84%)
(ii) No	17 (16%)
Type of surgery	
(i) PGS	64 (59%)
(ii) WGL	17 (15%)
(iii) USS	28 (26%)

**Table 3:** Percentage interobserver agreement (above —) and interobserver weighted kappa (below —) (*n* = 109).

Observers	Percentage interobserver agreement										BCCT.core
	1	2	3	4	5	6	7	8	9	10	
1	—	50	54	62	43	56	45	61	52	52	52
2	0.67	—	50	55	53	59	52	55	50	64	46
3	0.67	0.66	—	51	53	55	41	52	51	49	40
4	0.76	0.75	0.71	—	38	53	50	59	60	50	52
5	0.59	0.68	0.67	0.65	—	65	37	36	48	58	39
6	0.70	0.72	0.73	0.73	0.75	—	36	48	52	51	53
7	0.60	0.77	0.54	0.55	0.51	0.50	—	61	47	44	45
8	0.72	0.70	0.69	0.70	0.64	0.63	0.68	—	61	51	53
9	0.68	0.63	0.66	0.71	0.64	0.69	0.56	0.71	—	44	51
10	0.67	0.78	0.64	0.71	0.75	0.68	0.56	0.71	0.60	—	39
BCCT.core	0.50	0.54	0.52	0.65	0.55	0.61	0.52	0.61	0.59	0.50	—

			Interobserver weighted kappa								
